# Evaluating the PIRAA mechanism in undergraduate gerontological nursing: effects on knowledge, attitudes, and self-rated competencies

**DOI:** 10.1186/s12912-026-05209-3

**Published:** 2026-08-11

**Authors:** Zhouyuan Peng, Liyuan Sun, Yu Xu, Shuangyu Wang

**Affiliations:** 1 Medical School, Shenzhen University, Shenzhen, China; 2 Tianjin Medical University General Hospital, Tianjin, China

**Keywords:** Gerontological nursing, Educational innovation, Nursing students, Knowledge, Attitude, Competency, Aging, Older adults

## Abstract

**Background:**

Rapid population aging challenges health-care systems and underscores the need for educational models that build gerontological competencies in nursing students. The Policy–Industry–Research–Application–Academic (PIRAA) mechanism is a multifaceted pedagogical framework integrated into an undergraduate gerontological nursing course. This study evaluated the effectiveness of PIRAA in improving nursing students’ knowledge, attitudes, and self-rated competencies.

**Methods:**

A one-group pre–post, quasi-experimental design was used with 82 third-year undergraduate nursing students. PIRAA was delivered across 18 weeks via didactic sessions, a group workshop, and a nursing-home visit. Outcomes were assessed pre/post using the Chinese versions of Palmore’s Facts on Aging Quiz (FAQ), Kogan’s Attitudes toward Old People (KAOP) instrument, and a 32-item self-rated gerontological nursing competency scale. Paired-samples *t* tests evaluated within-person change.

**Results:**

FAQ correct rate increased from 0.48 ± 0.14 to 0.53 ± 0.11 (*t* = 3.31; *p* = 0.001). KAOP score increased from 109.12 ± 13.61 to 112.98 ± 14.03 (*t* = 2.47, *p* = 0.016). Self-rated competency total score increased from 90.87 ± 23.31 to 106.59 ± 20.16 (*t* = 6.59, *p* < 0.001), with gains across core knowledge, core skills, and role development.

**Conclusions:**

Embedding PIRAA in an undergraduate course was associated with measurable improvements in gerontological knowledge, attitudes, and self-rated competencies. Future research should employ randomized controlled trials to strengthen causal inference, assess durability through longitudinal follow-up, examine generalizability across multiple sites, and consider strategies for integration into national nursing curricula to promote sustainable implementation.

**Clinical trial number:**

Not applicable.

**Supplementary Information:**

The online version contains supplementary material available at https://doi.org/10.1186/s12912-026-05209-3.

## Background

The pace of population aging is accelerating rapidly, presenting significant challenges for health and social systems worldwide as they strive to adapt to this demographic shift [1]. In China, the situation is particularly pressing, with older adults aged 65 and over exceeding 200 million, marking the country’s entry into an aged society [2]. One of the most critical challenges of population aging is the growing discrepancy between the increasing demand for care for older adults and the limited workforce capacity to deliver it [3]. Consequently, the training of gerontological nursing personnel has become imperative.

Despite recent progress, gerontological nursing education in China still has substantial room for development [4]. Internationally, the preparation of gerontological nursing professionals has moved beyond the mere transmission of knowledge and skills [5]. Contemporary approaches place greater emphasis on shaping attitudes, strengthening humanistic values, and fostering self-awareness and professional identity formation. Consistent with this shift, European and North American competency frameworks stress career self-awareness, person-centered and aesthetic practice, and ethical decision-making in care for older adults [6], underscoring a global consensus that comprehensive gerontological education is essential for meeting the complex needs of older adults.

Current research and practical teaching experience highlight several persistent challenges in undergraduate gerontological nursing education. First, many younger students have limited understanding of population aging and little first-hand experience with aging, constraining their ability to adopt a comprehensive perspective and to appreciate the complex, specialized needs of older adults [7]. Second, the inherently interdisciplinary nature of gerontological nursing requires a broad repertoire of knowledge and competencies [8]. Third, prevailing pedagogies still underemphasize the translation of theory into practice, which is crucial for realizing students’ potential and sustaining long-term professional development. Accordingly, early, structured opportunities for problem identification, inquiry, and application should be embedded in the curriculum to strengthen theory–practice integration and contribute to the advancement of gerontological nursing.

Multiple pedagogical strategies and innovative educational designs have been employed to enhance undergraduates’ knowledge, attitudes, and competencies in gerontological nursing [9]. Beyond conventional lectures, approaches such as mindfulness-based empathy training [10], the Geriatric Medication Game [11], interprofessional education [12], simulation-based education [13], intergenerational service-learning [14], and institutional visits or clinical clerkships have been implemented to promote broader competence. Among these, simulation-based education is the most widely adopted, including aging-simulation suits [15], Mask-Ed™ simulation activities [16], daily life activities simulation [17], and virtual or hybrid simulation [18]. Evidence indicates that simulation-based education enhances nursing students’ empathy and attitudes toward older adults [19].

Although numerous pedagogical innovations have been reported, few comprehensive frameworks integrate policy, industry, research, application, and academic collaboration to systematically enhance student competencies. To address this gap, we developed the Policy–Industry–Research–Application–Academic (PIRAA) model–a comprehensive educational mechanism that integrates perspective-taking, practical ability, and innovative thinking to meet the needs of an aging society. This study evaluated the effectiveness of PIRAA in improving undergraduate nursing students’ knowledge, attitudes, and competencies in gerontological nursing. Our findings support the potential for broader implementation of PIRAA in undergraduate curricula and inform strategies to develop high-quality nursing professionals prepared for the challenges of population aging.

## Methods

### Study design and study population

This study employed a one-group pre-posttest quasi-experimental design. Randomization was not feasible in this educational setting because it would have required withholding course activities from students assigned to a control group. Accordingly, the intervention included the full cohort of third-year nursing undergraduates enrolled in one gerontological nursing course (*N* = 83). Post hoc power calculations based on observed effect sizes indicated power of 0.684 for knowledge, 0.683 for attitudes, and 0.995 for competencies, suggesting high power to detect large effects for competencies and moderate power for knowledge and attitudes.

### Study intervention: the PIRAA mechanism

PIRAA—an author-developed comprehensive educational mechanism—was embedded in an existing, mandatory gerontological nursing course that contributed to students’ grades and academic credit. The intervention spanned 18 weeks and included 15 didactic sessions, a group workshop (“Age-Friendly Home Environment Design”), and a nursing-home visit. PIRAA integrates complementary strategies to support perspective-taking, innovation, and practical competence. Mechanistic pathways and focal learning outcomes for each component are summarized in Figure S1 (logic model) and Table S1 (component-outcome mapping) in the Supplementary Materials.

**Policy (policy guidance):** The curriculum integrates national policy education and international perspectives into gerontological nursing. This approach aims to deepen students’ understanding of national policies and international trends while fostering professional responsibility and global awareness. A case library aligned with each chapter (policy, practice, and ethics) supports structured analysis and discussion. The teaching design specifies the content, methods, and timing of integrating policy/ideological elements, thereby strengthening contextual reasoning and critical thinking.

**Industry (innovation output):** To cultivate innovation and entrepreneurship, students participate in the “Maodie Joyland” social-inclusion project for older adults and people living with dementia. The project links classroom concepts with real-world problem-solving and encourages the development of practical, socially oriented solutions, reinforcing application-focused competencies.

**Research (nursing research):** Evidence-based thinking is emphasized throughout the course in the discussion of the textbook material and exposure to the relevant guidelines and latest research literature. A course WeChat account serves as a venue for student-authored briefs interpreting recent research and relevant policy updates in gerontological nursing, promoting critical reading, synthesis, and translation of evidence to practice.

**Application (practical application):** Community-based activities are incorporated to further reinforce the application of theoretical concepts to real-world settings. Using the “Maodie Joyland” mobile app, students conduct cognitive impairment screening, facilitate cognitive games, and deliver health education talks for older adults. These tasks reinforce assessment, communication, and management skills in gerontological care.

**Academic (education and teaching):** Practice-oriented teaching focuses on skill translation through technology-enhanced learning. Activities include training in age-friendly home environment modifications via a three–dimensional (3D) interactive platform with virtual reality (VR) goggles, and embodied simulation with an aging suit (e.g., kyphosis simulator, gloves, goggles, ear pods, cane) to simulate common age-related conditions such as limited range of motion, fine–motor deterioration, visual and hearing impairments, and mobility limitations.

Core PIRAA activities were embedded in the mandatory curriculum and delivered using standardized materials; completion of in-class activities was documented through routine course attendance and required assignment/report submissions, supporting comparable minimum exposure across students.

### Survey administration and data completeness

Outcomes were assessed using online surveys administered at two time points: within one week prior to the first course session (pre-test) and within one week after the final course session (post-test). Students completed the questionnaires independently (unsupervised). Survey distribution, data collection, export, and data cleaning were coordinated by a trained research assistant who was not involved in course teaching or student assessment; the instructor was not involved in survey administration to minimize social desirability bias. Although the course itself was compulsory, participation in the pre- and post-course surveys was entirely voluntary, and students could decline without any academic penalty. Students were explicitly informed that participation and responses would not affect course grades or evaluations and were encouraged to answer honestly. To minimize coercion, all activities were conducted within the regular curriculum, with no additional assignments or workload beyond standard course requirements. All 82 included participants completed both the pre- and post-course surveys, and there were no item-level missing data.

## Measures

### Demographic questionnaire

A structured questionnaire collected student characteristics, including age, gender, only-child status, whether nursing was the first choice in the National College Entrance Examination application, permanent residence (urban/rural), co-residence with older family members, perceived relationship with older family members, prior caregiving experience for older family members, participation in activities related to older adults, and willingness to work in care for older adults. These variables were collected because family structure, prior exposure to older adults, and contextual factors may shape baseline outcomes, particularly in the Chinese context.

### Palmore’s facts on aging quiz (FAQ)

Students’ knowledge of aging was assessed pre- and post-intervention using the validated Chinese version of Palmore’s Facts on Aging Quiz (FAQ) [20]. The FAQ contains 25 items with three response options (Yes/No/I do not know). Scores are reported as the percentage correct. The Chinese version has demonstrated acceptable reliability and validity (Cronbach’s α = 0.751; test–retest reliability = 0.982) [20].

### Kogan’s attitudes toward old people instrument (KAOP)

Attitudes toward older adults were measured with the Chinese version of Kogan’s Attitudes toward Old People (KAOP) [21] before and after the course. The instrument comprises two subscales—positive (11 items) and negative (14 items; reverse-scored). Items are rated on a 7-point Likert scale (1 = strongly disagree to 7 = strongly agree); total scores range from 25 to 175, with higher scores indicating more positive attitudes. The Chinese version has shown good psychometric properties (Cronbach’s α = 0.788; test–retest reliability = 0.974) [21].

### Gerontological nursing core competencies for undergraduate nursing students questionnaire

Changes in core competencies were assessed with a 32-item Chinese instrument developed for undergraduate gerontological nursing based on a core-competency evaluation framework [22]. The scale includes three subscales: core knowledge, core skills, and role development. Although the competency instrument includes a knowledge-related domain, it is conceptually distinct from the FAQ. The FAQ captures objective factual knowledge, whereas the competency scale captures self-perceived ability to apply knowledge in practice. This distinction mitigates risks of construct redundancy and inflated effect sizes. Items are rated on a 5-point Likert scale (1 = not capable to 5 = very capable); total scores range from 32 to 160, with higher scores reflecting higher levels of gerontological nursing competency. Internal consistency was excellent (Cronbach’s α = 0.986) [22]. Exploratory factor analysis in the present sample supported construct validity (Kaiser–Meyer–Olkin = 0.923; Bartlett’s test *p* < 0.001), identifying three factors that explained 76.7% of the variance, consistent with the theoretical structure. The original framework is available in Chinese; an English summary of domains and representative items is provided in Supplementary Material S2, and the full instrument can be obtained from the authors upon reasonable request.

### Statistical methods

Data analyses were conducted in IBM SPSS Statistics (version 26). Descriptive statistics, including frequencies, percentages, means, standard deviations, and totals, were calculated to summarize sample characteristics and scale scores. Between-group differences by demographic characteristics were examined using independent-samples *t* tests or one-way ANOVA, as appropriate. Within-subject change from pre- to post-intervention was assessed with paired-samples *t* tests. Assumptions of normality (Shapiro–Wilk) and homogeneity of variances (Levene’s test) were evaluated; all tests were two-tailed with α = 0.05. Where relevant, 95% confidence intervals are reported.

## Results

Of the 83 students initially recruited, one was excluded for invariant responses across all survey items, yielding a final sample of *N* = 82. Participants were aged 20–25 years (mean [standard deviation], 21.02 [0.93]); 70.7% (*n* = 58) were female. Most students (73.2%, *n* = 60) resided in urban areas; 20.7% (*n* = 17) were only children; and 67.1% (*n* = 55) did not co-reside with older family members. Detailed sociodemographic characteristics are shown in Table 1.

**Table 1:** Sociodemographic characteristics of nursing students (*N* = 82)

Variable	*N* (%) /mean (SD)
**Age**	21.02 (0.93)
**Gender**	
Male	24 (29.3)
Female	58 (70.7)
**Only child**	
Yes	17 (20.7)
No	65 (79.3)
**Residence** Rural/suburban Urban **Nursing as first choice in National College Entrance Exam application**	22 (26.8) 60 (73.2)
Yes	33 (40.2)
No	49 (59.8)
**Co-residence with older family members**	
Yes	27 (32.9)
No	55 (67.1)
**Relationship with older family members**	
Good	54 (65.9)
Normal	27 (32.9)
Not good	1 (1.2)
**Experience caring older family members**	
Yes	50 (61.0)
No	32 (39.0)
**Experience participating in activities related to older adults** Yes No	45 (54.9) 37 (45.1)
**Willingness to work in care for older adults** Yes No Indifferent	28 (34.1) 17 (20.7) 37 (45.1)

*SD Standard Deviation*

### Knowledge assessment scores

The mean correct rate on the FAQ increased significantly from 0.48 ± 0.14 at baseline to 0.53 ± 0.11 post–intervention (*t* = 3.312, *p* = 0.001), reflecting a small but statistically significant gain in students’ knowledge about aging (Table 2). Posttest item analysis showed that several misconceptions remained common; the lowest correct rates were observed for items such as “At least one in ten older adults lives in long–term care institutions,” “Most older people report rarely feeling bored,” and “Older adults become more religious with age.” In bivariate analyses, no significant differences in knowledge were observed by sociodemographic characteristics.

**Table 2:** Pre– and post–intervention comparisons of knowledge, attitudes, and competencies scores in nursing students (*N* = 82)

Variable	Pre–test	Post–test	Mean difference (95% CI)	t	*p*
Knowledge (FAQ)	0.48 ± 0.14	0.53 ± 0.11	0.05 (0.01, 0.09)	3.312	**0.001**
Attitudes (KAOP)	109.12 ± 13.61	112.98 ± 14.03	3.86 (0.74, 6.96)	2.465	**0.016**
Competencies (core knowledge)	39.02 ± 10.63	46.30 ± 9.40	7.28 (4.11, 10.45)	6.477	**< 0.001**
Competencies (core skills)	25.45 ± 6.93	29.78 ± 6.36	4.11 (2.09, 6.13)	5.807	**< 0.001**
Competencies (role development)	26.39 ± 6.85	30.50 ± 5.64	4.33 (2.28, 6.38)	5.506	**< 0.001**
Competencies (total score)	90.87 ± 23.31	106.59 ± 20.16	15.72 (8.87, 22.57)	6.591	**< 0.001**

FAQ Facts on Aging Quiz, KAOP Kogan’s Attitudes toward Old People

### Attitude assessment scores

The mean KAOP score increased from 109.12 ± 13.61 to 112.98 ± 14.03 (*t* = 2.465, *p* = 0.016), indicating more positive attitudes toward older adults after taking the course (Table 2). The results of the independent *t* tests and ANOVA indicated that gender (*t* = − 2.015, *p* = 0.047), participation in activities related to older adults (*t* = 2.025, *p* = 0.046), and willingness to work in care for older adults (*t* = 3.743, *p* = 0.028) were associated with more positive attitudes. No other sociodemographic characteristics were significantly associated with attitudes.

### Competency assessment scores

Students’ gerontological nursing competencies showed greatest gains, with mean total scores increasing from 90.87 ± 23.31 to 106.59 ± 20.16 (*t* = 6.591, *p* < 0.001; Table 2). Significant improvements were observed across all three subscales—core knowledge (*t* = 6.477, *p* < 0.001), core skills (*t* = 5.807, *p* < 0.001), and role development (*t* = 5.506, *p* < 0.001)—indicating a broad-based enhancement of competency. Despite these overall gains, some items remained relatively low post-intervention, particularly those involving higher-order, context-dependent abilities: (i) engaging with professional gerontological organizations to advocate for the inclusion of disease prevention and end–of–life care in national legislation, regulations, and reimbursement; (ii) contributing to the development of quality standards for care across hospitals, communities, families, and nursing homes; (iii) applying strategies to improve older adults’ treatment adherence during care transitions, and (iv) evaluating how different reimbursement systems affect the accessibility and affordability of geriatric services. In bivariate analyses, no significant differences in self-rated competencies were observed by sociodemographic characteristics.

## Discussion

In this study, we implemented the PIRAA mechanism—an integrated pedagogical approach that leverages modern teaching technologies to deepen understanding of aging, strengthen interdisciplinary competence, and enhance knowledge application competency—within a gerontological nursing course and evaluated its effects on students’ knowledge, attitudes, and self-rated competencies.

Students entered the course with moderate baseline knowledge and showed measurable post-intervention gains, consistent with prior work [23]. The median pretest FAQ correct rate (0.48; IQR 0.40–0.60) exceeded that reported among Chinese nurses in 2011–2012 (0.40; IQR 0.32–0.52) [20] but was lower than that observed among Hong Kong nursing students (0.60 ± 0.11) [24]. These discrepancies may reflect differences in participant seniority and the maturity of gerontological nursing education across regions. In mainland China, recent curricular reforms driven by rapid population aging may partly explain the relatively higher baseline scores observed in this study [25], while the Hong Kong cohort comprised senior students who had completed a gerontological nursing course. Although knowledge accuracy improved significantly, the magnitude was modest, with only a 0.05 increase, smaller than the gain reported in an Iranian study using an aging-simulation suit [23]. A plausible explanation is the markedly longer exposure in that study (approximately two hours per student) versus about 30 min in our course. Future iterations should allocate more time for experiential components to maximize learning effects. In addition, the persistence of high error rates on several items highlights the need to reinforce content on gerontological psychology and care continuity within the curriculum.

Female students, those with prior experience caring for older adults, and those willing to work in gerontological care exhibited more positive attitudes, consistent with previous findings [26, 27]. Post-intervention, attitudes improved significantly, aligning with results from studies using aging-simulation suits [28] and intergenerational service-learning [29]. By contrast, a study employing standardized patients found no meaningful attitudinal change [30], suggesting that simulation-based learning, along with direct contact with older adults, may be important for shifting attitudes. While both simulation and real-world interactions in our course may have contributed to these gains, our design does not permit definitive attribution to specific components. A meta-analysis has reported that simulation-based learning is among the most effective approaches for improving nursing students’ attitudes toward older adults [31]. Conversely, a systematic review of qualitative studies noted that, although simulation can enhance empathy, it may inadvertently reinforce stereotypes, whereas practical experiences more effectively increase willingness to work in care for older adults [32]. More recent reviews further indicate that, although many interventions reduce negative attitudes, they rarely increase willingness to enter gerontological care [33, 34]. By integrating both simulation and practice, the PIRAA model addresses limitations of single-method interventions, aiming not only to improve attitudes but also to cultivate sustained interest in gerontological nursing. Nevertheless, willingness to work in gerontological care is multifactorial and shaped by structural and cultural factors (e.g., job prestige, remuneration, public perceptions). As highlighted by a recent systematic review [33], future research should examine how educational approaches interact with system-level incentives and professional recognition to attract graduates into the field.

After the semester-long gerontological course, students’ competencies improved significantly on the total score and all subscales of the competency questionnaire. Consistent with our findings, Korea’s IGNITE program—a multimodal initiative incorporating experiential learning, critical reflection, and emerging technologies (e.g., care-assistive robots and simulation)—reported comparable gains in gerontological competence [34], suggesting that multifaceted, technology-enhanced approaches may enhance competence. Evidence from the United States [30] and Uganda [35] likewise shows that experiential and community-based models can meaningfully strengthen applied competence, creating opportunities to translate theory into practice and to develop clinical reasoning, communication, and decision-making in care for older adults. Nonetheless, post-intervention competency scores in our cohort remained mid-range. Low-scoring items clustered in higher-order, context-dependent domains (e.g., policy advocacy, care-transition adherence, and financing analysis). This pattern likely reflects limited practical depth: such competencies typically require sustained, longitudinal engagement, and—despite the industry and application elements within PIRAA—an 18-week exposure may be insufficient to achieve the necessary experiential breadth and depth.

The PIRAA mechanism was associated with improvements in nursing students’ knowledge, attitudes, and self-rated competencies in gerontological care, aligning with global trends that emphasize competency-based education for aging populations. For instance, Germany’s Geriatric Nursing Care Training Regulations prioritize career self-awareness, whereas the United States and Canada emphasize evidence-informed practice, cultural competence, and prevention of elder abuse [6]. Similarly, Australia proposed a national competency framework that highlights ethical decision-making and interprofessional collaboration [36]. By combining theoretical instruction with experiential activities (e.g., simulation, community engagement, and innovation projects) PIRAA reflects these priorities and offers a potential model that can strengthen China’s nursing education while contributing to international discourse on preparing a workforce capable of meeting the challenges of population aging.

Despite advances within China and elsewhere, broader international sharing of approaches in gerontological nursing education remains needed [6]. Implementation barriers may arise in resource-limited settings, where components such as VR equipment, aging suits, and interdisciplinary projects can be difficult to deploy. Lower-cost alternatives—such as 3D videos, classroom role-playing, or simple tools (e.g., weighted clothing, blurred-vision goggles, earplugs) to simulate aging experiences—can be considered. Interdisciplinary learning can also be introduced in phases, beginning with small-scale collaborations before expanding to larger projects. Such phased and flexible adaptations can help ensure that PIRAA remains feasible and transferable across diverse educational contexts.

Although this study offers useful insights into applying the PIRAA mechanism in undergraduate gerontological nursing education, several limitations should be noted. First, in the absence of a control group, the observed improvements cannot be attributed solely to PIRAA; gains may also reflect within-semester progression, learning from parallel coursework, or other out-of-class educational influences. Second, competencies were assessed by self-report, which may diverge from actual performance; future studies should incorporate objective, performance-based assessments. Third, while in-class tasks and graded assignment reports were structured to ensure comparable participation, platform use beyond class was open and unmonitored; out-of-class engagement was therefore not quantified, potentially introducing unmeasured exposure heterogeneity. Finally, the single-site sample from one Chinese university may limit generalizability to other programs and settings.

## Conclusions

PIRAA yielded measurable gains in knowledge, attitudes, and self-rated competencies and may serve as a scalable model for competency-based gerontological education in China and beyond. However, additional optimization is needed to support higher levels of mastery. Further implementation across cohorts and settings will enable iterative refinement of PIRAA and inform context-appropriate adaptation for broader adoption. Future research should employ controlled designs where feasible, incorporate objective performance-based assessments, and evaluate longer-term outcomes across multiple sites and regions.

## Supplementary Information

Below is the link to the electronic supplementary material.

Supplementary Material 1

Supplementary Material 2

Supplementary Material 3

## Data Availability

The datasets used and/or analyzed during the current study are available from the corresponding author on reasonable request.

## Abbreviations

PIRAA: Policy–Industry–Research–Application–Academic
3D: Three–dimensional
VR: Virtual reality
FAQ: Facts Aging Quiz
KAOP: Kogan’s Attitudes toward Old People
ANOVA: Analysis of variance
IQR: Interquartile range
