# Supplementary material for: Evaluating the PIRAA mechanism in undergraduate gerontological nursing: effects on knowledge, attitudes, and self-rated competencies
Source: BMC Nurs. 2026 Aug 11;25:1083. doi: 10.1186/s12912-026-05209-3 (PMC13741752; doi:10.1186/s12912-026-05209-3)
Supplement: Supplementary file 1 [file 12912_2026_5209_MOESM2_ESM.pdf]

Supplementary Material S2. English summary of the Chinese gerontological nursing competency framework and representative items

Purpose. To facilitate international comparison and reproducibility, this file provides an English summary of the competency framework underlying the 32-item self-rated gerontological nursing competency scale and a set of representative items. Because access to some Chinese databases may be restricted for international readers and because full reproduction of proprietary scales may raise copyright concerns, we present domains, scoring, and representative items; the full Chinese instrument can be obtained from the published sources and/or from the authors upon reasonable request.

Terminology note. The cited Chinese source's English title uses the term "geriatric"; in this manuscript we use "gerontological nursing" consistently because the framework domains cover comprehensive nursing care for older adults across settings.

Framework domains and scoring

The scale contains 32 items across three domains:

- Core Skills (9 items)
- Core Knowledge (15 items)
- Role Development (8 items)

Items are rated on a 5-point Likert scale (1 = not capable to 5 = very capable). Total score ranges from 32 to 160; higher scores indicate higher self-rated competency in gerontological nursing.

Representative items – Core Skills

**Representative item (English translation)**
Recognize reasons for sensory changes and atypical behaviors in older adults and apply an individualized care philosophy in practice.
Use evidence-based approaches and reliable tools to assess physical health, functional status, mental health, and social well-being in older adults.
Assess how living environments (home, community, nursing home, hospital, etc.) influence the health of older adults.
Develop health education plans based on older adults' needs and provide health guidance and skills training to older adults and their families.

Representative items – Core Knowledge

**Representative item (English translation)**
Implement health promotion measures to prevent or reduce risk factors leading to functional decline, reduced quality of life, and disability in older adults.

Apply strategies to improve treatment adherence in older adults and support care transitions across home, hospital, and nursing home settings.
Apply ethical and legal principles to address complex ethical and legal issues in the care of older adults.
Assess how reimbursement systems affect access, availability, and affordability of care for older adults.

Representative items – Role Development

**Representative item (English translation)**
Encourage older adults to actively participate in all aspects of their health care.
Participate in developing quality standards for gerontological care across hospitals, communities, homes, and nursing homes.
Engage with professional organizations to promote inclusion of disease prevention and end-of-life care in legislation, regulation, and reimbursement systems.
Identify researchable questions in gerontological nursing practice and design and implement research projects.

Original Chinese sources

1. Li Y, Song J. Construction of the core competency system for undergraduate nursing students in geriatric nursing. Chinese Journal of Practical Nursing. 2015;31(8):549–552. DOI: 10.3760/cma.j.issn.1672-7088.2015.08.002.
2. Zhang B, Zhang Y, Wang R, et al. Status quo of geriatric nursing core ability among practice nurses and influencing factors. Chongqing Medicine. 2018;47(33):4274–4277. DOI: 10.3969/j.issn.1671-8348.2018.33.019.

Copyright and use note. This English summary is provided as supplementary material for peer review and scholarly comparison. The original instrument and publications remain the property of their respective authors/publishers.
