# Supplementary material for: Evaluating the PIRAA mechanism in undergraduate gerontological nursing: effects on knowledge, attitudes, and self-rated competencies
Source: BMC Nurs. 2026 Aug 11;25:1083. doi: 10.1186/s12912-026-05209-3 (PMC13741752; doi:10.1186/s12912-026-05209-3)
Supplement: Supplementary file 2 [file 12912_2026_5209_MOESM3_ESM.pdf]

Table S1 Week-by-Week PIRAA Program Structure

Week	Contact hours	Theme	PIRAA	Teaching methods	Expected outcomes	Assessment / Evidence
1	1.5	Introduction to aging & course orientation	Policy, Academic	Lecture; guided case discussion	Attitudes; Knowledge	Pre-test (FAQ/KAOP/competency)
2	1.5	Aging changes & gerontological theories (I)	Policy, Research	Lecture; case discussion, WeChat brief	Knowledge	WeChat brief
3	1.5	Aging changes & gerontological theories (II)	Policy, Research	Lecture; case discussion, WeChat brief	Knowledge	
4	1.5	Health assessment of older adults (I)	Industry, Application	Lecture, "Maodie Joyland" feature-proposal task	Competencies	Feature proposal (assessment module)
5	1.5	Health assessment of older adults (II)	Industry, Application	Lecture, "Maodie Joyland" feature-proposal task	Competencies	

6	1.5	Daily care for older adults	Policy, Research	Lecture, case discussion	Knowledge, Competencies	Home mod report（Standardized task sheet and facilitator guide used across groups.）
7	1.5	Age-friendly home environment design	Policy, Academic	Group workshop：aging-suit simulation (~30 min) + structured briefing/debriefing and reflection (~15 min)+VR/3D interactive training (~45 min). Delivered in small groups (≈3–4 students/group) under facilitator supervision	Attitudes, Knowledge, Competencies	
8	1.5	Common health problems & care (I)	Policy, Research	Lecture, case discussion WeChat brief	Knowledge, Competencies	WeChat brief
9	1.5	Common health problems & care (II)	Policy, Research	Lecture, case discussion WeChat brief	Knowledge, Competencies	
10	0	Holiday	—	—	—	—

11	1.5	Common diseases & care (I)	Policy, Research	Lecture, case discussion<br>WeChat brief	Knowledge, Competencies	WeChat brief
12	1.5	Common diseases & care (II)	Policy, Research	Lecture, case discussion<br>WeChat brief	Knowledge, Competencies	
13	1.5	Mental health & spiritual care (I)	Industry, Application	Lecture, case discussion<br>"Maodie Joyland" game-design task	Attitudes, Competencies	Game design concept
14	1.5	Mental health & spiritual care (II)	Industry, Application	Lecture, case discussion<br>"Maodie Joyland" game-design task	Knowledge, Competencies	
15	3	Health promotion & community practice	Industry, Application	Nursing-home visit:<br>Facility tour and orientation (1 h)+ Health education session for residents and community-dwelling older adults (1 h)+ Cognitive screening/assessment using the "Maodie Joyland" mini-program (1 h)<br>Delivered in small groups (≈6–8 students/group) under facilitator supervision	Attitudes, Competencies	Participation log; screening count; talk outline（Standardized task sheet and facilitator guide used across groups.）

16	1.5	Safe medication use	Policy, Research	Lecture, case discussion<br>WeChat brief	Knowledge, Competencies	WeChat brief
17	1.5	End-of-life care	Policy, Research	Lecture, case discussion	Attitudes, Competencies	
18	1.5	Elder abuse & protection of rights	Policy, Research	Lecture, case discussion	Attitudes, Competencies	Post-test (FAQ/KAOP/competency)

FAQ Facts on Aging Quiz, KAOP Kogan's Attitudes toward Old People
